# The Potential of Glucosinolates and Their Hydrolysis Products as Inhibitors of Cytokine Storms

**DOI:** 10.3390/molecules29204826

**Published:** 2024-10-11

**Authors:** Kingsley Ochar, Kanivalan Iwar, Vadakkemuriyil Divya Nair, Yun-Jo Chung, Bo-Keun Ha, Seong-Hoon Kim

**Affiliations:** 1 Council for Scientific and Industrial Research, Plant Genetic Resources Research Institute, Bunso P.O. Box 7, Ghana; ocharking@korea.kr; 2 National Agrobiodiversity Center, National Institute of Agricultural Sciences, Rural Development Administration, Jeonju 54874, Republic of Korea; kani05@korea.kr; 3 Department of Plant Sciences, Central University of Himachal Pradesh, Shahpur Campus, Kangra District, Shahpur 176206, HP, India; divyanair013@hpcu.ac.in; 4 National Creative Research Laboratory for Ca^2+^ Signaling Network, Jeonbuk National University Medical School, Jeonju 54896, Republic of Korea; yjchong@jbnu.ac.kr; 5 Department of Applied Plant Science, Chonnam National University, Gwangju 61186, Republic of Korea

**Keywords:** cytokine storm, enzymatic hydrolysis, glucosinolates, NF-κB, TNF-α

## Abstract

A cytokine storm is an intense inflammatory response characterized by the overproduction of proinflammatory cytokines, resulting in tissue damage, and organ dysfunction. Cytokines play a crucial role in various conditions, such as coronavirus disease, in which the immune system becomes overactive and releases excessive levels of cytokines, including interleukins, tumor necrosis factor-alpha (TNF-α), and interferon-gamma (IFN-γ). This anomalous response often leads to acute respiratory distress syndrome (ARDS), disseminated intravascular coagulation (DIC), and multiple organ injury (MOI). Glucosinolates are plant secondary metabolites predominantly found in *Brassica* vegetables, but are also present in other species, such as *Moringa* Adens and *Carica papaya* L. When catalyzed by the enzyme myrosinase, glucosinolates produce valuable products, including sulforaphane, phenethyl isothiocyanate, 6-(methylsulfinyl) hexyl isothiocyanate, erucin, goitrin, and moringin. These hydrolyzed products regulate proinflammatory cytokine production by inhibiting the nuclear factor kappa-light-chain-enhancer of activated B-cell (NF-κB) signaling pathway and stimulating the nuclear factor erythroid 2-related factor 2 (*Nrf2*) signaling pathway. This action can alleviate hyperinflammation in infected cells and modulate cytokine storms. In this review, we aimed to examine the potential role of glucosinolates in modulating cytokine storms and reducing inflammation in various conditions, such as coronavirus disease. Overall, we found that glucosinolates and their hydrolysis products can potentially attenuate cytokine production and the onset of cytokine storms in diseased cells. In summary, glucosinolates could be beneficial in regulating cytokine production and preventing complications related to cytokine storms.

## 1. Introduction

Cytokines are a diverse group of small proteins (<40 kDa) that are produced and secreted by almost all cells. They are functionally involved in facilitating intercellular communication, regulating immune responses, and controlling cell growth and specialization [1]. They also play a role in regulating angiogenesis, which is the physiological process by which new blood vessels are formed from pre-existing vessels [2]. For instance, in patients with coronavirus disease 2019 (COVID-19), the causative virus induces acute production and unrestrained discharge of proinflammatory and anti-inflammatory cytokines that mediate immunopathogenesis [3,4]. This unrestrained release of proinflammatory cytokines further activates immune cells, resulting in the amplified production and release of more cytokines [1]. This feedback loop leads to hyperactivation of the immune system and elevated production and release of many proinflammatory cytokines. Key cytokines involved in this process include interleukins (ILs), tumor necrosis factor-alpha (TNF-α), interferons (IFNs), colony-stimulating factors (CSFs), chemokines, lymphokines, and monokines [5]. Specific treatments can also cause inflammatory syndrome by triggering an excessive release of cytokines from immune cells [1]. This dysregulated immune response induces a severe condition known as cytokine release syndrome or cytokine storm. In patients with COVID-19, a cytokine storm typically occurs in pulmonary tissues [6]. However, it involves diverse interrelated events that eventually cause multiple organ failure and ultimately result in death [7,8]. A cytokine storm is a common clinical condition in various diseases and conditions, such as anaphylaxis, graft-versus-host disease (GVHD), acute respiratory distress syndrome (ARDS), and systemic inflammatory response syndrome (SIRS) [6,9]. In particular, ARDS is a life-threatening condition that has caused death in many patients with COVID-19. Over the past years, ARDS has caused many deaths worldwide. However, so far, no specific treatment is practically available to cure COVID-19-related conditions in critically ill patients [10]. Nevertheless, various medically recommended immunotherapeutic approaches can suppress or modulate the proinflammatory receptors (markers) involved in the weakening of the human repertory systems [11]. In particular, they can increase the adaptive immune response by producing cytokines and chemokines, which are the major drivers of inflammation associated with respiratory conditions and innate immunity [12]. For instance, previous studies have revealed the association of TNF-α and several IL-related cytokines with lung damage in patients with ARDS [6,11]. Hence, there is an increasing interest in exploring various approaches to inhibit receptors associated with ARDS and reduce cytokine storms in diseases like COVID-19 [13].

In recent years, several natural compounds distributed in plant species have received significant research attention, especially for exploring their potential applications as novel products targeting receptors linked to acute viral infections, such as those caused by severe acute respiratory syndrome coronavirus (SARS-CoV), severe acute respiratory syndrome coronavirus 2 (SARS-CoV-2), and Middle East respiratory syndrome coronavirus (MERS-CoV), in humans [14,15]. Diverse natural products or metabolites are present in plants [16]; they can be classified into primary and secondary metabolites [17,18]. Primary metabolites are directly involved in cellular processes and play essential roles in plant growth and development [19]. On the other hand, secondary metabolites are organic substances that are directly involved in plant defense and resistance mechanisms. In plants, secondary metabolites not only facilitate cellular adaptation to diverse physiological stress responses but also play vital roles in strengthening defense against herbivores and enhancing disease control [20]. In humans, secondary metabolites exhibit several beneficial effects, such as antioxidant, anti-inflammatory, anticancer, and antidiabetic activities [21]. Consequently, they have several applications, e.g., as food additives, food flavoring agents, and nutraceuticals [22]. Among these, glucosinolates (GSLs), a large group of nitrogen-containing secondary metabolites predominantly found in *Brassica* plants, have received extensive research attention because of their diverse biological properties and health benefits [23,24,25]. They are derived from different amino acid precursors and are classified into aliphatic, aromatic, and indolic compounds. These metabolites are widely distributed throughout the plant body, including leaves, stems, flowers, roots, and seeds [24]. In humans, GSLs interact with several receptors involved in various chronic diseases, such as cancer, hypertension, and diabetes [26]. GSLs are biologically activated through the enzymatic hydrolysis of myrosinase, thereby producing active substances, such as thiocyanates and isothiocyanates (ITCs) [27]. These hydrolysis products exhibit promising health-promoting effects, including antibacterial, anticancer, anti-inflammatory, antioxidant, cardioprotective, and immunomodulatory effects, in humans [24]. The dietary intake of GSL-rich foods can prevent inflammation and oxidative stress, primarily because of the involvement of GSLs in activating detoxification enzymes, scavenging reactive oxygen species (ROS), and eliciting immune functions [26,28]. The essential roles of GSLs in modulating cytokine storms in patients with COVID-19 have also been explored. Bahoosh et al. [29] recently reported that the low mortality rate observed in patients with COVID-19 in Eastern Asia is partly attributed to their high consumption of cruciferous vegetables, commonly prepared as fermented cabbage (kimchi), known to have a high GSL content. These fermented products may enhance nuclear factor erythroid 2-related factor 2 (*Nrf2*)-associated pathways, thereby attenuating the severity of COVID-19 symptoms [30]. Kimchi prepared using cabbage and many other vegetables also exhibits various beneficial effects against insulin resistance-associated diseases, such as diabetes [31,32], cardiovascular diseases [33], and dyslipidemia [34], as well as aging [35]. Kimchi fermented for a long period can reduce insulin intolerance to a greater extent than fresh products [36]. In addition to their essential contribution to the strong flavor of *Brassica* vegetables, ITCs interact with several biological pathways, such as *Nrf2* and nuclear factor kappa-light-chain-enhancer of activated B-cell (NF-κB) pathways, thereby modulating immune responses in humans [29]. These interactions inhibit NF-κB activation and enhance the activity of *Nrf2*, thereby reducing the expression of cytokines [37,38]. In this review, we aimed to provide a comprehensive summary of research advances on the potential role of GSLs and their hydrolysis products in moderating cytokine storms in patients with COVID-19.

## 2. Cytokines and Cytokine Storms

**Figure 1:**
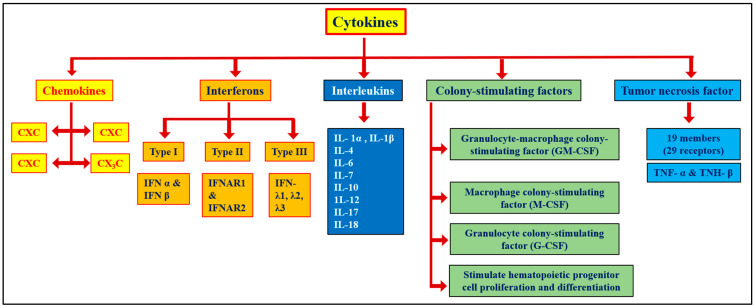
Types of cytokines (Tisoncik et al., 2012) [2].

Cytokines are a diverse group of small proteins released by cells to facilitate intercellular communication (Figure 1 and Table 1). These proteins include ILs, TNF-α, CSF, chemokines, monokines, and lymphokines [5]. Chemokines represent a subgroup of small cytokines that participate in regulating immunological reactions [39]. The excessive release of chemokines induces ARDS and drives hyperinflammation, particularly in patients with COVID-19. ARDS typically occurs in critically ill patients with COVID-19 and is characterized by swelling of the lungs, accumulation of fluid in the alveoli, and subsequent damage to the protective membrane of the alveoli [40]. This alveolar membrane damage enables fluid to leak into the alveoli, resulting in the inability of the lungs to pass oxygen to the bloodstream [41]. Many viruses, such as SARS-CoV, MERS-CoV, influenza virus, and SARS-CoV-2, often cause infections by elevating chemokine levels. For instance, Qudus et al. [39] found that chemokines have severe clinical consequences in patients with COVID-19 and can even cause death in patients with critical conditions. Various chemokines, such as IL-8, C-X-C motif chemokine ligand 2 (CXCL2), and C-X3-C motif chemokine ligand 1 (CX3CL1), play essential roles in attracting macrophages to the lungs, while chemokine ligand 2 (CCL2), CCL3, CCL7, and CCL8 facilitate inflammatory responses. Hyperactivation of the immune system consequently leads to the onset of a cytokine storm [42,43]. The term cytokine storm was initially used by Chatenoud [44] to describe a condition induced by the monoclonal antibody muromonab-CD3 during immunosuppressive therapy for solid organ transplantation. Later, Ferrara [45] used the term cytokine storm to describe an acute GVHD occurring during engraftment syndrome following allogeneic stem cell transplantation. Cytokine storms are intense immune responses that can cause significant tissue injury, leading to multiple organ dysfunction. This reaction involves the rapid release of high levels of proinflammatory cytokines, such as IL-6, TNF-α, and IL-1β, which are associated with various illnesses, including viral infections (e.g., COVID-19), autoimmune conditions, and sepsis [46]. ARDS is a severe clinical condition linked to cytokine storms in patients with COVID-19 [6]. The management of cytokine storms in clinical settings presents a significant challenge, highlighting the need for developing potent inhibitors to mitigate their adverse effects. The effective suppression of cytokine storms could significantly reduce morbidity and mortality associated with hyperinflammatory conditions.

## 3. Pathophysiology and Management of Cytokine Storms in COVID-19

**Figure 2:**
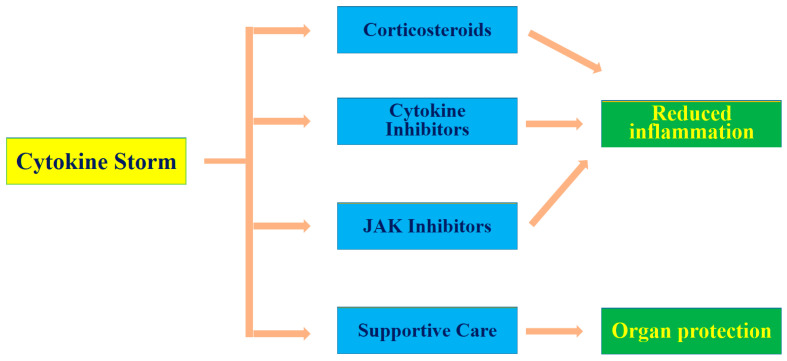
Feedback loop of cytokine production and therapeutic management strategies.

Less than half a decade ago, the novel and deadly viral disease COVID-19 was categorized as a pandemic by the World Health Organization (WHO) [59,74,75]. This disease, caused by SARS-CoV-2, is distinct from previous coronavirus diseases such as SARS and MERS, reported in 2002 and 2012, respectively [76,77]. SARS-CoV-2 is a highly contagious virus [59,76]. In terms of geographic distribution and the number of people infected, this novel zoonotic virus has been described as one of the most rapidly spreading pathogens ever recorded in human history [78]. A key clinical feature of COVID-19 is the abnormal production of various cytokines after the pathogen attacks immune cells [51]. The mechanism underlying cytokine storms involves interactions among several immune cells, such as macrophages, dendritic cells, and T cells, and the inflammatory cytokines they produce [42]. These cells produce various inflammatory cytokines, such as IL-6, TNF-α, IL-1β, and IFN-γ [79]. These cytokines further recruit and stimulate more immune cells, establishing a cycle that can induce uncontrollable inflammation. During the early phases of an infection or immune reaction, cytokines play a crucial role in regulating the body’s defense mechanisms [79]. However, the overproduction of cytokines can result in increased vascular permeability, swelling, low blood pressure, and disseminated intravascular coagulation, ultimately causing organ damage, dysfunction, or failure [42]. Recent research has highlighted the significance of endothelial cells and the coagulation system in enhancing the impact of cytokine storms, which can cause various complications, such as ARDS and septic shock [80]. Figure 2 presents a summary of the feedback loop of cytokine production and various therapeutic management strategies. In clinical settings, various therapeutic approaches are used to reduce inflammation and protect organs. These therapeutic strategies include the use of corticosteroids [81], cytokine inhibitors [82], Janus kinase (JAK) inhibitors [83], and supportive care [84] (Figure 2). While clinical evidence on the efficacy of these treatment strategies continues to accumulate, other natural alternatives that may exhibit broad-spectrum anti-inflammatory activity need to be explored [85].

The initial infection trigger leads to a cascade of immune responses, resulting in severe inflammation and tissue/organ damage due to dysregulation. Corticosteroids, such as dexamethasone, are commonly used to dampen the overall inflammatory response. Cytokine inhibitors, which specifically target key cytokines (such as IL-6), help reduce cytokine-driven inflammation. Janus kinase (JAK) inhibitors block the signaling pathways activated by cytokines, further mitigating their effects. Supportive care, including oxygen therapy and fluid management, plays a crucial role in stabilizing patients and supporting organ function.

## 4. GSL Hydrolysis Products as Potential Modulators of Cytokine Storms

**Figure 3:**
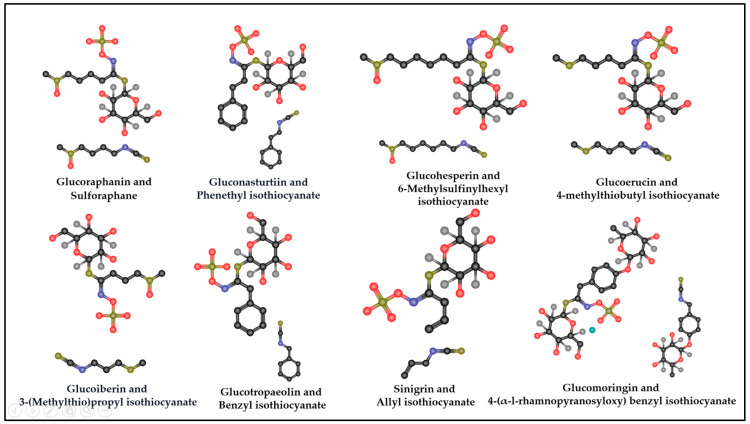
Molecular structure of glucosinolates and their hydrolysis products.

**Figure 4:**
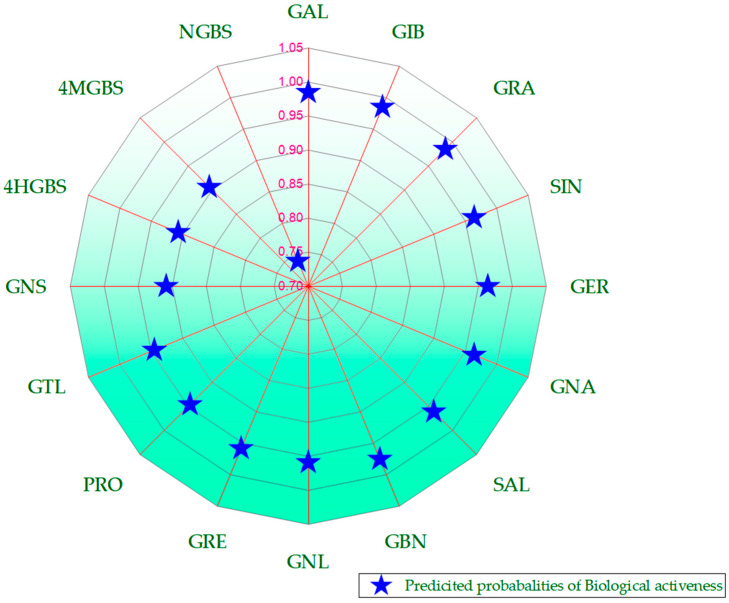
Therapeutic potential of representative glucosinolates, predicated using Way2Drug (https://www.way2drug.com/passonline) accessed on 5 July 2024. The predicted probabilities of biological activity were high (>0.7), suggesting that the glucosinolates possess significant properties that could effectively modulate inflammatory responses associated with cytokine storms. GAL: Glucoalyssin; GIB: glucoiberin; GRA: glucoraphanin; SIN: sinigrin; GER: glucoerucin; GNA: gluconapin; SAL: sinalbin; GBN: glucobrassicin; GNL: gluconapoleiferin; GRE: glucoraphenin; PRO: progoitrin; GTL: glucotropaeolin; GNS: gluconasturtiin; 4HGBS: 4-hydroxiglucobrassicin; 4MGBS: 4-methoxiglucobrassin; and NGBS: neoglucobrassicin.

As consumer demand for healthy and functional food products continues to rise, the intake of fruits and vegetables has consistently been encouraged because of their widely valued nutrient content and health-promoting phytochemical content [86]. Secondary metabolites, including phenolics, terpenoids, and nitrogen-containing compounds, are important sources of anti-inflammatory, antioxidant, and antitumor agents that enhance the body’s defense against several chronic diseases [18]. GSLs are the most abundantly expressed phytochemicals in the Brassicaceae family of plants. They have also been profiled in a few other plants, such as moringa [87,88,89] and papaya [90,91,92]. Enzymatic hydrolysis is a crucial step in the transformation of GSLs into various useful products. In their raw state, when plant tissues, such as leaves, roots, seeds, and flowers, are disrupted through processes like cutting or chewing, GSLs are hydrolyzed by endogenous myrosinase. The hydrolysis process produces aglucone intermediates [93]. The unstable aglucone readily and rapidly rearranges into biologically active or stable compounds, including thiocyanates, ITCs, nitriles, epithionitriles, hydroxynitriles, oxazolidine-2-thiones, and indole-3-carbinols [25]. ITCs include sulforaphane (SFN), phenethyl ITC (PEITC), 6-(methylsulfinyl) hexyl ITC (6-MSITC), erucin (ER), and moringin (Figure 3) [92]. ITCs are involved in plant defense against herbivores and pathogens [94]. In terms of human nutrition and health, GSLs and their corresponding ITCs play diverse roles in modulating the effects of proinflammatory cytokines (Figure 4, Table 2) [94]. Their essential contribution to preventing various chronic diseases has increased research interest over the years [95,96,97]. Given their anti-inflammatory properties, ITCs have the potential to counteract the onset and progression of cytokine storms, which are a major cause of death in patients with COVID-19 [98,99,100]. The relative production of GSLs significantly varies within and between species because of the variability of GSL side chains resulting from enzymatic hydrolysis [97].

### 4.1. Glucoraphanin and Its Hydrolysis Product, SFN

The hydrolysis of glucoraphanin produces SFN, which regulates various biological pathways associated with diverse diseases, including COVID-19 [108]. SFN interacts with and represses the activity of NF-κB, a complex network leading to the inhibited or reduced production and secretion of proinflammatory cytokine markers [113,114,115]. SFN also participates in the *Nrf2* pathway, where it activates *Nrf2* to inhibit the production of proinflammatory cytokines (e.g., IL-6 and IL-8) by different cells, such as macrophages and monocytes [116,117,118]. SFN is a potent *Nrf2* inducer and suppressor of NF-κB gene expression, as observed in COVID-19 lung biopsies; thus, it contributes to improving patient outcomes [119]. Kiser et al. [120] found that SFN activates and inhibits *Nrf2* and NF-κB pathways, respectively, which may promote positive outcomes in patients with COVID-19. These properties of SFN may lower the progression of cytokine storms [113]. In a recent study, Gasparello et al. [121] investigated the effects of SFN on SARS-CoV-2 infection and NF-κB-related genes implicated in COVID-19 cytokine storms. They found that SFN inhibits the replication of SARS-CoV-2 and reduces viral RNA production in infected cells by stabilizing an inactive NF-κB/SFN/DNA complex. This phenomenon lowers NF-κB levels and inhibits the expression of proinflammatory genes encoding IL-1β and IL-8; however, its effect on IL-6 remains inconclusive. Similarly, in an in vitro study, Ordonez et al. [122] demonstrated the antiviral effects of SFN against SARS-CoV-2 and HCoV-OC43 infections. They used mice as model organisms and found that prophylactic SFN administration significantly reduced viral load, lung injury, and immune cell activation while modulating the inflammatory response [122]. In similar studies, SFN was found to inhibit NLRP3 inflammasome activation without the involvement of the *Nrf2* pathway, thereby modulating cytokine storms in patients with COVID-19 [123,124]. This effect was attributed to decreased and increased miR-155 and miR-223 expression levels, respectively [125]. The SARS-CoV-2 N protein could directly induce the activation of the NLRP3 inflammasome, indicating that the inhibitory effect of SFN on this pathway could reduce inflammation in patients with COVID-19. Moreover, Chen et al. [126] recently found that SFN modulates cytokine storms in patients with COVID-19 by inhibiting the SARS-CoV-2 3CLpro enzyme through a slow-binding, mixed-type mechanism. They observed that the inhibitory effect of SFN against the SARS-CoV-2 3CLpro enzyme could significantly reduce viral replication, thereby decreasing the viral load and the associated immune response that contributes to cytokine storms in patients with COVID-19. By stabilizing the SFN–3CLpro complex and inhibiting the protease activity essential for viral maturation, SFN interferes with the ability of the virus to propagate, in turn limiting the excessive release of proinflammatory cytokines. This reduction in cytokine production can help prevent a severe cytokine storm, a crucial factor in response to severe inflammation in patients with COVID-19. The role of glucoraphanin in reducing symptoms like nasal congestion, cough, and fatigue has been previously reported, suggesting the potential of SFN in reducing the onset and process of cytokine storms in SARS-CoV-2 infections [29]. These findings suggest the potential of SFN as a therapeutic agent for managing cytokine storms in various conditions, such as coronavirus infections.

### 4.2. Gluconasturtiin and Its Hydrolysis Product, PEITC

Several studies have demonstrated the essential role of PEITC, derived from gluconasturtiin, in moderating inflammation, cell proliferation, and oxidative stress in chronic human diseases [127,128]. PEITC regulates the activity of inflammatory markers, such as IL-1β, cyclooxygenase-2 (COX-2), and inducible nitric oxide synthase (iNOS) by interacting with glutathione (GSH), thereby reducing oxidative stress and inflammation [129,130]. Similar to SFN, PEITC is involved in the *Nrf2*/ARE pathway. In particular, it modulates the production and translocation of *Nrf2*, which is responsible for upregulating the expression of antioxidant and detoxification enzymes [131]. This mechanism may help lower the occurrence and severity of cytokine storms [132]. Moreover, similar to SFN, PEITC interacts with the NF-κB pathway by inhibiting the phosphorylation of key components, such as inhibitory kappa B kinase beta (IκKβ) and IκBα [115]. This inhibition prevents the transcriptional activation of NF-κB-controlled genes, such as those encoding vascular endothelial growth factor (VEGF), cyclin D1, and B-cell lymphoma-extra large (Bcl-XL), which are involved in cell proliferation and survival [131,133]. By blocking these processes, PEITC effectively reduces inflammatory responses and cellular stress, potentially attenuating the severity of conditions associated with chronic inflammation, as well as cytokine storms.

### 4.3. Glucohesperin and Its Hydrolysis Product, 6-MSITC

The compound 6-MSITC is an important product obtained through the enzymatic hydrolysis of glucohesperin, found in *Brassica* species, including wasabi and rocket salad [134]. Previous studies on chronic disease conditions have revealed the potential interaction between 6-MSITC and many inflammatory and cell proliferation-associated genes, such as *Nrf2*, iNOS, and COX-2 genes, which modulate the production of various proinflammatory cytokines [111,135]. For instance, *Nrf2* can repress inflammation-related genes in cells known to produce proinflammatory cytokines, such as IL-1β [136]. Moreover, the *Nrf2* signaling pathway can control tissue damage in various pathogen infections and is important for promoting immune response to viral infections and inflammation in patients with COVID-19 [119]. Okamoto et al. [134] reported that 6-MSITC exhibits significant anti-inflammatory and anticoagulant activities, particularly in endothelial cells. They found that 6-MSITC reduces the production of proinflammatory cytokines, such as IL-6 and TNF-α, and lowers the adhesion of immune cells to endothelial cells, which are crucial factors in the development and progression of inflammation and cytokine storms in patients with COVID-19 [137]. By modulating these inflammatory pathways and maintaining vascular homeostasis, 6-MSITC could potentially alleviate severe vascular inflammation and clotting complications associated with cytokine storms in COVID-19.

### 4.4. Glucoerucin and Its Hydrolysis Product, Erucin

Glucoerucin (4-methylsulfanylbutyl) is a well-studied GSL in *Brassica* species, such as broccoli sprouts, pak choy, and Chinese cabbage. The enzymatic hydrolysis of glucoerucin produces ER, a dietary ITC [138]. Structurally, ER and SFN are related; both have an aliphatic side chain and may share common pharmacokinetic properties [139]. In vivo studies have revealed that ER and glucoraphanin-derived SFN products can be interconverted and have the potential to suppress inflammatory cytokines, thereby helping to reduce cytokine storms in patients with COVID-19 [97,140]. The biological mechanisms of action of erucin include the regulation of ROS induction, tumor suppression and apoptosis, and cell cycle arrest [141]. ER plays modulatory roles in influencing phase I (Cyp450), phase II [quinone reductase (QR) and glutathione transferase (GST)], and phase III detoxification [139,142]. The antioxidant, anti-proliferative, and anti-inflammatory effects of ER in relation to the therapeutic control of chronic diseases have been reported in several studies [143,144]. ER can inhibit the production and release of proinflammatory cytokines, including IL molecules (IL-1β and IL-12), by obstructing the NF-κB pathway; NF-κB is usually present in elevated levels in COVID-19 infections [145,146]. ER suppresses the transcription of several proinflammatory markers, such as TNF-α, IL-1β, and IL-12, suggesting its potential in regulating cytokine storms and consequently managing COVID-19 [29].

### 4.5. Glucoiberin and Glucoiberverin and Their Hydrolysis Products, Iberin and Iberverin, Respectively

Iberin and iberverin are products of GSL hydrolysis; they are classified as ITCs and are derived from glucoiberin and glucoiberverin, respectively. Similar to their SFN homolog, iberin and iberverin can trigger *Nrf2*-dependent gene expression [107]. This activation leads to the reduced production of proinflammatory enzymes and cytokine markers, which is crucial for regulating cytokine storms in patients with COVID-19 [29]. In a recent study, Hosokawa et al. [147] examined the potential of iberin as an anti-inflammatory agent in human oral epithelial cells. They found that iberin repressed the expression of IL-6, CXCL10, vascular cell adhesion molecule-1 (VCAM-1), iNOS, and COX-2 in TNF-α-stimulated TR146 cells. Moreover, iberin administration downregulated the activation of NF-κB, STAT3, and p70S6 kinase (p70S6K)-S6 ribosomal protein (S6) pathways. Iberin also reduced inflammatory mediator expression in human oral epithelial cells by preventing the activation of specific signal transduction pathways. These properties of the SFN homolog could potentially play a role in modulating cytokine storms in patients with COVID-19 by inhibiting the excessive production of inflammatory cytokines and chemokines, reducing the activation of inflammatory pathways, and enhancing antioxidant defenses.

### 4.6. Glucotropaeolin and Their Hydrolysis Product, Benzyl ITC (BITC)

Glucotropaeolin, an aromatic GSL, undergoes enzymatic hydrolysis to produce BITC, which exhibits anti-inflammatory properties by interacting with the NF-κB signaling pathway [29]. BITC downregulates NF-κB, a key modulator of inflammatory responses associated with cytokine storms. It also participates in the defense mechanism in response to oxidative stress. By neutralizing oxidative stress and attenuating inflammation, BITC helps manage cytokine storms.

### 4.7. Sinigrin and Its Hydrolysis Product, Allyl ITC (AITC)

Sinigrin, an aliphatic glucosinolate, and its hydrolyzed product, AITC, play essential roles as therapeutic agents against inflammation, cancer, oxidative stress, and microbial infections [148]. The characteristic anti-inflammatory properties of AITC suggest its potential role in regulating cytokine storms. As an ITC, AITC can inhibit NF-κB-mediated inflammatory signaling by suppressing TNF-α and IL-6 expression resulting from the transcription of NF-κB, thereby alleviating cytokine storms [149]. Caglayan et al. [104] revealed the ability of AITC to offset oxidative stress and inflammation by modulating *Nrf2*/HO-1 and NF-κB pathways in patients with traumatic brain injury. They found that AITC substantially reduced the expression levels of proinflammation cytokines, such as IL-β and IL-6. Among others, the expression level of *Nrf2* was significantly increased, resulting in the suppression of NF-κB. The involvement of AITC in these pathways suggests that it is a promising candidate for extenuating cytokine storms and improving inflammatory and oxidative stress-related conditions.

### 4.8. Glucomoringin and Its Hydrolysis Product, Moringin

Although glucosinolates are predominantly synthesized in the Brassicaceae family of plants, they have also been profiled in other plant species, such as *Moringa oleifera* Lam. The GSL glucomoringin is abundant in moringa seeds and leaves [150]. Myrosinase-mediated hydrolysis of glucomoringin produces moringin [4-(α-L-rhamnosyloxy) benzyl ITC], functionally implicated in mitigating oxidative stress [151,152]. Moringin increases the expression of *Nrf2* and represses the NF-κB signaling pathway [153], suggesting its prospects as an antioxidant and anti-inflammatory agent and a modulator of cytokine storms. Moreover, moringin can suppress the production and release of proinflammatory cytokines, such as TNF-α [154].

## 5. Conclusions

A cytokine storm is an anomalous immune response that is markedly prominent in COVID-19 infections. The hydrolysis products of certain GSLs are promising compounds for modulating cytokine storms because of their ability to modulate inflammatory responses. Upon hydrolysis by myrosinase, an endogenous enzyme, GSLs produce useful metabolite products, such as SFN, PEITC, erucin, AITC, and BITC. These products exhibit strong anti-inflammatory properties by inhibiting the NF-κB pathway and activating the *Nrf2* signaling pathway, thereby reducing the production of proinflammatory cytokines. This synergistic action is vital in offsetting the severity of cytokine storms, as seen in COVID-19. Although emerging evidence supports the potential of GSLs in attenuating cytokine storms, further experimental research is essential. For instance, molecular docking simulations are warranted to identify specific molecular targets of GSL-derived compounds. Enhanced understanding and development of these compounds could significantly improve treatment strategies for inflammatory diseases and infections characterized by cytokine dysregulation. A notable limitation of this review is the lack of comprehensive evidence confirming the efficacy of GSLs and their hydrolysis products in mitigating cytokine storms. Although initial studies suggest promising benefits, further research is essential to fully elucidate their therapeutic potential. Future investigations should prioritize rigorous mechanistic and translational research to bridge the gap between laboratory findings and practical applications, ultimately advancing our understanding of the role of GSLs in immune modulation and cytokine regulation.

## Figures and Tables

**Table 1:** Cytokine markers related to cytokine storms.

Cytokine/Chemokine	Outcome	Cell Source	Function	Reference
Interleukin-1α (IL-1α) or pro-IL-1α	Proinflammatory cytokine	Activated macrophages, epithelial cells, endothelial cells, neutrophils, monocytes, fibroblasts, mesenchymal cells, necrotic cells, and dendritic cells	1. Initiates and triggers the secretion of other inflammatory cytokines, such as IL-6 and TNF-α. 2. Contributes to tumor development in humans. 3. Activates T lymphocytes (causes fever), producing INF and IL-2 required for immune response. 4. Plays a role in tissue repair and remodeling in chronic obstructive pulmonary disease.	[39,47,48,49]
Interleukin-1β (IL-1β) or pro-IL-1β	Proinflammatory cytokine	Macrophages, monocytes, fibroblasts, neutrophils, epithelial cells, endothelial cells, dendritic cells, activated T cells, and T-helper 1 (Th1) cells	1. Activates immune response and promotes fever. 2. Activates Th1 cells. 3. Induces IL-6 production. 4. Mediates inflammatory response, which is crucial for host response and resistance to pathogens. 5. Aggravates tissue damage under chronic and acute disease conditions. 6. Acts as a major cytokine in many rheumatic disorders.	[5,39,50]
Interleukin-1 receptor antagonist (IL-1Ra)	Anti-inflammatory cytokine	Hepatic cells, monocytes, macrophages, neutrophils, keratinocytes, epithelial cells, and fibroblasts	Limits IL-1-mediated inflammation.	[50,51,52,53]
Interleukin-18 (IL-18) or IFN-γ-inducing factor (IGIF)	Proinflammatory cytokine	Monocytes, macrophages, keratinocytes, mesenchymal cells, endothelial cells, osteoblasts, epithelial cells, and dendritic cells	1. Stimulates IL-6 and IFN-γ expression. 2. Controls neutrophil function. 3. Mediates adaptive immune responses by regulating the activities of several cytokines. 4. Together with IL-2, IL-18 enhances the production of Th2 cytokines, such as IL-3, IL-4, IL-9, and IL-13. 5. Together with IL-3, IL-18 stimulates the production of IL-4, IL-13, and histamine by various cells, such as mast cells and basophils. 6. Stimulates the production of other cytokines, such as IL-1β, IL-6, IL-17, and TNF-α; C-reactive protein (CRP); and chemokines that are essential for host innate defense and cytokine storms. 7. Overexpression enhances the accumulation of inflammatory cells, such as neutrophils, eosinophils, cytotoxic T lymphocytes (CD8 + T cells), and macrophages, in lung injury and fibrosis. 8. Aberrant expression can be induced by constant NLRP3 inflammasome activity, resulting in the increased production of downstream inflammatory mediators, such as IFN-γ, IL-6, and TNF-α, involved in cytokine storms. 9. Abundant presence in the lungs relative to other tissues increases the activity of IL-18 and the likelihood of a cytokine storm. 10. Induces inflammatory and cytotoxic immune cell activities, leading to autoimmunity. 11. Together with IL-12, IL-18 enhances natural killer (NK) cell activities in response to cancers and infections.	[5,39,54,55,56]
Interleukin-33 (IL-33)	Proinflammatory cytokine	Myofibroblasts, endothelial cells, epithelial cells, and active fibroblasts	1. Activates various immune subgroups. 2. Promotes type 2 immune response. 3. Promotes pulmonary fibrosis. 4. Reduces IFN-γ expression and increases IL-4, IL-5, and IL-13 expression. 5. Elevated levels can increase joint inflammation through the increased activation of neutrophils and mast cells, in addition to the increased expression of inflammatory cytokines, such as IL-6 and IL-1β. 6. Increases the production of type 2 cytokines, such as IL-4, IL-9, IL-10, IL-13, and tumor growth factor-beta (TGF-β). 7. Regulates ARDS-induced inflammation by mediating the secretion of IL-13 by T-reg cells. 8. Participates in the mechanism of fibrosis.	[39,57]
Interleukin-36α (IL-36α)	Proinflammatory cytokine	Bronchial epithelium, monocytes, macrophages, keratinocytes, dendritic cells, brain tissue, gut, and skin	1. Induces inflammatory responses by stimulating NF-κB and mitogen-activated protein kinases (MAPKs). 2. Abnormally activates IL-36 signaling to enhance inflammatory conditions.	[58]
Interleukin-36β (IL-36β)	Proinflammatory cytokine	Bronchial epithelium, monocytes, macrophages, keratinocytes, dendritic cells, brain tissue, gut, and skin	1. Induces inflammatory responses by activating NF-κB and MAPK via the intracellular signaling cascade. 2. Abnormal activation promotes inflammatory conditions detrimental to various organs, such as the kidneys, lungs, and intestines.	[58]
Interleukin-36γ (IL-36γ)	Proinflammatory cytokine	Bronchial epithelium, monocytes, macrophages, keratinocytes, dendritic cells, brain tissue, gut, and skin	1. Induces inflammatory responses by activating NF-κB and MAPK via the intracellular signaling cascade. 2. Abnormal activation increases inflammatory conditions that may be detrimental to various organs, such as the lungs, kidneys, and intestines. 3. Exhibits both proinflammatory and anti-inflammatory properties.	[58]
Interleukin-37 (IL-37)	Anti-inflammatory cytokine	Monocytes, dendritic cells, macrophages, leukocytes, tonsil B cells, plasma cells, natural killer cells, stimulated B lymphocytes, and epithelial cells	1. Powerful anti-inflammatory cytokine capable of countering a broad spectrum of proinflammatory cytokines, such as IL-1β, TNF, and IFN-γ, as well as TLRs. 2. Transduces anti-inflammatory signals by activating Mer–PTEN–DOK pathways, while suppressing NF-κB and MAPK pathways. 3. Promotes innate and acquired immunity and increases aging-related immunosenescence. 4. Represses tumor angiogenesis and metastasis.	[58,59,60,61]
Interleukin-4 (IL-4)	Anti-inflammatory cytokine	Mast cells, T-helper (Th1 and Th2) cells, and basophils	1. Mediates Th2 cell-mediated synthesis of various cytokines (IL-4, IL-5, IL-6, and IL-13) and Th2 immune response. 2. IL-4 and IL-13 are largely associated with the remodeling of fibrogenic inflammation. 3. Involved in the activation of the JAK–STAT pathway. 4. Inhibits proinflammatory monocyte-related cytokines, such as IL-8, IL-6, IL-1, and TNF-α. 5. Suppresses the cytotoxic activity of macrophages. 6. Promotes antibody production.	[50,51,62]
Interleukin-6 (IL-6)	Proinflammatory cytokine	Macrophages, T cells, and fibroblasts	1. Facilitates immune response. 2. Induces and releases other proinflammatory cytokines (promotes inflammation). 3. Acts as an antibody and pyrogen potentiator (enhances the sensitization of antibodies). 4. Plays a crucial role in cytokine storms. 5. Increases vascular permeability. 6. Causes lymphocyte exhaustion and necrosis.	[39,50,51]
Interleukin-12 (IL-12)	Proinflammatory cytokine	Dendritic cells and macrophages	Enhances cellular immune response.	[50]
Interleukin 13 (IL-13)	Anti-inflammatory cytokine	T cells, mast cells, basophils, and activated Th2 cells	1. Downregulates inflammation and supports tissue repair. 2. Associated with the remodeling of fibrogenic inflammation. 3. Similar to IL-4, IL-13 is a key participant in the T2 pathway. 4. Coordinates with IL-4 to activate M2 macrophages.	[50,51,62]
Interleukin-17 (IL-17)	Proinflammatory cytokine	Macrophages, neutrophils, Th17 cells, and iNKT cells	1. Acts as a proinflammatory cytokine. 2. Promotes neutrophilic inflammation, stimulating cytokine production.	[39]
Interleukin-10 (IL-10)	Anti-inflammatory cytokine	Macrophages, regulatory T cells, monocytes, Th1 cells, and Th2 cells	1. Downregulates immune response and suppresses inflammation. 2. Suppresses Th1 cell-mediated cytokine release. 3. Negatively regulates T cells by increasing cytotoxic CD8+ T cells.	[39,50]
Tumor necrosis factor-alpha (TNF-α)	Proinflammatory cytokine	Macrophages, monocytes, epithelial cells, dendritic cells, Th1 cells, Th17 cells, CD8+ T cells, and endothelial cells	1. Promotes vascular permeability, enhances autoimmune response, and induces a proinflammatory state. 2. Improves IL-6 production and functions as a pyrogenic cytokine. 3. Acts as a principal participant in cytokine storm interactions.	[39,51]
Interleukin 8 (IL-8) or C-X-C motif chemokine ligand 8 (CXCL8)	Proinflammatory chemokine	Macrophages, monocytes, endothelial cells, fibroblasts, activated T cells, Th1 cells, neutrophils, epithelial cells, and hepatocytes	1. Acts as a proinflammatory cytokine. 2. Controls Th1 and Th2 responses. 3. Elevated concentrations enhance ARDS and increase the severity of pneumonia or COVID-19. 4. Acts as a neutrophil chemotactic agent that recruits neutrophils to infected tissues, increasing the severity of COVID-19. 5. Associated with many disorders, such as severe lung damage in SARS-CoV and MERS-CoV infections.	[39,63]
C-X-C motif chemokine 10 ligand (CXCL10)/IP-10	Proinflammatory chemokine	Dendritic cells, Th2 cells, and macrophages	1. Acts as a chemoattractant for immune cells. 2. Activates Th1 cell function. 3. Binds to the CXCR3 receptor and recruits various cells, such as macrophages, Th1 cells, and NK cells. 4. Regulates cellular angiostasis, proliferation, apoptosis, and chemotaxis.	[39]
C-X-C motif chemokine ligand 1 (CX3CL1), also known as fractalkine (FKN)	Inflammatory chemokine	Vascular endothelial cells	1. Recruits mononuclear phagocytes. 2. Causes neurological vascular damage and thrombosis. 3. Induces endothelial dysfunction, causing atherosclerosis, cardiovascular diseases, and other complications. 4. Acts as an invasion signal, attracting mononuclear phagocytes to the lungs. 5. Facilitates thrombosis and vascular damage.	[39]
Chemokine ligand 2 (CCL2)/Monocytechemoattractant protein-1 (MCP-1)	Inflammatory chemokine	Monocytes, endothelial cells, epithelial cells, fibroblasts, astrocytes, microglial cells, and mesangial cells	1. Facilitates communication between lymphocytes and myeloid cells. 2. Activates Th1 cell function. 3. Regulates the movement, infiltration, and differentiation of monocytes to macrophages, thereby determining the body’s response to inflammation. 4. High levels upregulate the expression of IL-1, IL-6, TNF-α, matrix metalloproteinase-8, and intercellular adhesion molecule-1, causing inflammation. 5. Increased levels aggravate neurological conditions, such as stroke, Alzheimer’s disease, and dementia. 6. Amplified expression increases the chances of developing kidney failure and worsens prognosis in patients with severe COVID-19.	[39]
Granulocyte-macrophage colony-stimulating factor (GM-CSF)	Inflammatory chemokine	Epithelial cells, monocytes, activated T cells, and macrophages	1. Serves as a chemoattractant for immune cells. 2. Activates Th1 cell function. 3. Enhances proinflammatory cytokines and chemokines, influencing Th17 responses. 4. Significantly enhances GM-CSF-activated immune cells.	[39]
Interferon-γ-inducible protein (IP10)	Inflammatory chemokine	Monocytes, neutrophils, endothelial cells, keratinocytes, fibroblasts, mesenchymal cells, dendritic cells, astrocytes, hepatocytes, and leukocytic eosinophils	1. Secreted in response to IFN-γ and plays a key role in the inflammatory response. 2. Both amplifies and suppresses the proliferation of different cells and receptors. 3. Promotes cancer cell proliferation. 4. Elevated levels cause uncontrolled inflammatory responses, induce tissue damage, and facilitate the development of ARDS, leading to a cytokine storm.	[51,64]
Monocyte chemoattractant protein-1 (MCP-1), also known as chemokine (CC-motif) ligand 2 (CCL2)	Inflammatory cytokine	Macrophages, monocytes, fibroblasts, epithelial cells, endothelial cells, smooth muscle cells, astrocytes, and microglial cells	1. Plays a vital role in the inflammation process by attracting other inflammatory cells, such as monocytes, T cells, macrophages, and dendritic cells, to the site of inflammation or infection and enhancing their expression. 2. Elevated levels are associated with chronic inflammatory and respiratory failure in patients with COVID-19.	[65]
Interferon-gamma (IFN-γ)	Proinflammatory cytokine	Macrophages, T cells, NK cells, and cytotoxic T lymphocytes	1. Exhibits both anti- and protumorigenic effects. 2. Stimulates the activation of immune response and elimination of pathogens. 3. Prevents the overactivation of the immune system and tissue damage. 4. Promotes the activation of macrophages, thereby enhancing their ability to kill intracellular pathogens. 5. Promotes Th1/Th2 cell differentiation, which is important for cell-mediated immunity.	[51,66,67]
Interleukin-15 (IL-15)	Proinflammatory cytokine	Macrophages, monocytes, epithelial cells, dendritic cells, keratinocytes, epidermal skin cells, fibroblasts, bone marrow stromal cells, and nerve cell lymphocytes	1. Promotes the survival, proliferation, and activities of NK cells. 2. Acts as a potent autocrine regulator of proinflammatory cytokine production. 3. High concentrations are suitable for TNF-α, IL-1, and IL-6 production, while very low concentrations favor IL-10 production. 4. Stimulates neutrophil movement and function, e.g., the secretion of IL-8, via NF-κB activation. 5. Induces the proliferation of mast cells in the absence of IL-15Rα and the β chain. 6. Maintains the balance of induced inflammatory cytokines and the homeostatic responses of natural killer and CD8+ T cells. 7. Stimulates cytotoxic CD8+ T cell and NK cell responses. 8. Supports lymphocyte homeostasis by contributing to the overall maintenance of lymphoid cell populations in the body.	[68,69]
Interleukin-21 (IL-21)	Anti- and proinflammatory cytokine	T cells, natural killer cells, and stromal cells	1. Lowers the expression of IL-6 and TNF-α, thereby reducing inflammatory proteins involved in cytokine storms. 2. Promotes the development of B cells and plasma cells and upregulates IgG1 antibody formation. 3. Stimulates the proliferation of CD8 effector cells and costimulates the proliferation and function of T and NK cells.	[69,70]
Interleukin-2 (IL-2)	Proinflammatory cytokine	Activated T cells, Th1 cells, dendritic cells, and lymphocytes	1. Promotes the development of T cells, B cells, natural killer (NK) cells, and innate lymphoid cells (ILCs) associated with the survival or death of immune cell populations. 2. Regulates white blood cells, especially lymphocytes. 3. Participates in the activation of T cells to produce TNF-α and IFN-γ, particularly in response to antigen recognition. 4. Influences the survival and differentiation of cells and negatively regulates immune activation.	[51,71,72]
Interleukin-7 (IL-7)	Proinflammatory cytokine	Keratinocytes, dendritic cells, hepatocytes, neurons, epithelial cells, bone marrow stromal cells, and fibroblasts	1. Promotes the growth, survival, proliferation, and differentiation of B and T cells. 2. Maintains the survival and homeostasis of naive and memory T cells in the peripheral immune system.	[73]

**Table 2:** Representative glucosinolates, mechanisms of action of their hydrolysis products, and potential effects in regulating cytokine storms.

Glucosinolate	Hydrolysis Product	Mechanism of Action	Effect on Cytokine Storm	Reference
Glucoraphanin	Sulforaphane and sulforaphane nitrile	Downregulates NF-kB translocation and Bax and caspase 3 expression.	Reduces the production of proinflammatory cytokines (e.g., IL-1β) and apoptosis.	[101,102]
Sinigrin	Allyl isothiocyanate (AITC)	1. Regulates the NF-κB-mediated inflammatory cascade. 2. Inhibits the production of IL-6 and TNF-α by downregulating NF-κB-mediated transcription. 3. Upregulates the *Nrf2* pathway via the suppression of NF-κB.	1. Reduces the expression levels of proinflammatory markers, such as IL-1β and IL-6. 2. Increases antioxidative mechanisms. 3. Demonstrates other therapeutic effects, such as anticancer, antimicrobial, and wound-healing effects.	[103,104,105]
Gluconasturtiin	Phenethyl isothiocyanate (PEITC)	1. Influences *Nrf2* expression and nuclear translocation. 2. Inhibits the transcription of NF-κB.	Reduces the secretion of proinflammatory mediators, exhibiting anti-inflammatory properties.	[106]
Glucoiberin	Iberin and iberverin	Induces the *Nrf2*-dependent gene expression signaling pathway.	Stimulates immune cell activity.	[29,107]
Glucoiberverin	Iberin and iberverin	A homolog of sulforaphane that can induce *Nrf2*-dependent gene expression.	Stimulates immune cell activity.	[29,107]
Glucotropaeolin	Benzyl isothiocyanate (BITC)	Downregulates NF-κB signaling.	Possesses anti-inflammatory properties.	[37,103]
Glucoerucin	Erucin	Directly inhibits the NF-κB signaling pathway by activating *Nrf2* to inhibit IL-6, IL-8, and IL-12 expression.	Possesses anti-inflammatory properties, reducing the transcription of proinflammatory molecules.	[108,109]
Progoitrin	Goitrin	Reduces TNF-α secretion.	Induces an anti-inflammatory response.	[29,110]
Epiprogoitrin	Goitrin	Reduces TNF-α secretion.	Induces an anti-inflammatory response.	[29,110]
Glucohesperin	6-(Methylsulfinyl) hexyl isothiocyanate (6-MSITC)	1. Regulates COX-2, iNOS, TNF-α, IL-1β, and IL-6 gene transcription. 2. Modulates *Nrf2* activity within the nucleus.	Possesses anti-inflammatory, antimicrobial, and anticancer properties.	[111]
Glucomoringin	Moringin	1. Interacts with *Nrf2* to inhibit oxidative stress 2. Exhibits better inhibitory effects against NF-kB activity than SFN.	Exhibits a broad range of biological effects by counteracting inflammation, antioxidative stress, tumorigenesis, and microbial infections.	[29,112]

## Abbreviations

3CLpro, 3-chymotrypsin-like protease; AITC, allyl isothiocyanate; ARDS, acute respiratory distress syndrome; ARE, antioxidant response element; Bcl-XL, B-cell lymphoma-extra large; BITC, benzyl isothiocyanate; CCL2, chemokine ligand 2; COVID-19, coronavirus disease 2019; COX-2, cyclooxygenase-2; CRP, C-reactive protein; CRS, cytokine release syndrome; CTLs, cytotoxic T lymphocytes; CSF, colony-stimulating factor; CX3CL1, C-X-C motif chemokine ligand 1; CXCL10, C-X-C motif chemokine ligand 10; CXCR3, C-X-C motif chemokine receptor 3; DOK, downstream of tyrosine kinase/docking protein; GST, glutathione transferase; GVHD, graft-versus-host disease; IFN, interferon; IKKβ, Inhibitory kappa B kinase beta; iNKT cells, invariant natural killer T cells; iNOS, inducible nitric oxide synthase; ITC, isothiocyanate; JAK, Janus kinase; *Nrf2*, nuclear factor erythroid 2-related factor 2; NLR, nucleotide-binding domain, leucine-rich repeat; NLRP3, nucleotide-binding domain, leucine-rich repeat family pyrin domain containing 3; MAPK, mitogen-activated protein kinase; MCP-1, monocyte chemoattractant protein-1; MERS-CoV, Middle East respiratory syndrome coronavirus; PEITC, phenethyl isothiocyanate; PTEN, phosphatase and tensin homolog; SARS-CoV, severe acute respiratory syndrome coronavirus; SARS-CoV-2, severe acute respiratory syndrome coronavirus 2; SFN, sulforaphane; SIRS, systemic inflammatory response syndrome; STAT3, signal transducer and activator of transcription; TBI, traumatic brain injury; TGF-β, tumor growth factor-beta; TLRs, toll-like receptors; TNF-α, tumor necrosis factor-alpha; VCAM-1, vascular cell adhesion molecule-1; and VEGF, vascular endothelial growth factor.

## References

[1] Kany S., Vollrath J.T., Relja B. (2019). Cytokines in inflammatory disease. Int. J. Mol. Sci..

[2] Tisoncik J.R., Korth M.J., Simmons C.P., Farrar J., Martin T.R., Katze M.G. (2012). Into the eye of the cytokine storm. Microbiol. Mol. Biol. Rev..

[3] Jiang Y., Rubin L., Peng T., Liu L., Xing X., Lazarovici P., Zheng W. (2022). Cytokine storm in COVID-19: From viral infection to immune responses, diagnosis and therapy. Int. J. Biol. Sci..

[4] Ahmed A.A., Ad’hiah A.H. (2021). Interleukin-37 is down-regulated in serum of patients with severe coronavirus disease 2019 (COVID-19). Cytokine.

[5] Lopez-Castejon G., Brough D. (2011). Understanding the mechanism of IL-1β secretion. Cytokine Growth Factor Rev..

[6] Montazersaheb S., Hosseiniyan Khatibi S.M., Hejazi M.S., Tarhriz V., Farjami A., Ghasemian Sorbeni F., Farahzadi R., Ghasemnejad T. (2022). COVID-19 infection: An overview on cytokine storm and related interventions. Virol. J..

[7] Vinayagam S., Sattu K. (2020). SARS-CoV-2 and coagulation disorders in different organs. Life Sci..

[8] Zhou Z., Ren L., Zhang L., Zhong J., Xiao Y., Jia Z., Guo L., Yang J., Wang C., Jiang S. (2020). Heightened innate immune responses in the respiratory tract of COVID-19 patients. Cell Host Microbe.

[9] Rudd J.M., Tamil Selvan M., Cowan S., Kao Y.-F., Midkiff C.C., Narayanan S., Ramachandran A., Ritchey J.W., Miller C.A. (2021). Clinical and histopathologic features of a feline SARS-CoV-2 infection model are analogous to acute COVID-19 in humans. Viruses.

[10] Rathi M., Singh P., Bi H.P., Shivanna A., Kavadichanda C., Tripathy S.R., Parthasarathy J., Tota S., Maurya S., Vijayalekshmi V. (2021). Impact of the COVID-19 pandemic on patients with systemic lupus erythematosus: Observations from an Indian inception cohort. Lupus.

[11] Que Y., Hu C., Wan K., Hu P., Wang R., Luo J., Li T., Ping R., Hu Q., Sun Y. (2022). Cytokine release syndrome in COVID-19: A major mechanism of morbidity and mortality. Int. Rev. Immunol..

[12] Bergamaschi C., Terpos E., Rosati M., Angel M., Bear J., Stellas D., Karaliota S., Apostolakou F., Bagratuni T., Patseas D. (2021). Systemic IL-15, IFN-γ, and IP-10/CXCL10 signature associated with effective immune response to SARS-CoV-2 in BNT162b2 mRNA vaccine recipients. Cell Rep..

[13] Hu B., Huang S., Yin L. (2021). The cytokine storm and COVID-19. J. Med. Virol..

[14] Kolodnitsky A., Ionov N., Gravel I., Poroikov V. (2023). Natural compounds from medicinal plants against COVID-19. Explor. Drug Sci..

[15] Raghav A., Khan Z.A., Upadhayay V.K., Tripathi P., Gautam K.A., Mishra B.K., Ahmad J., Jeong G.-B. (2021). Mesenchymal stem cell-derived exosomes exhibit promising potential for treating SARS-CoV-2-infected patients. Cells.

[16] Tzvetkov N.T., Kirilov K., Matin M., Atanasov A.G. (2024). Natural product drug discovery and drug design: Two approaches shaping new pharmaceutical development. Nephrol. Dial. Transplant..

[17] Salam U., Ullah S., Tang Z.-H., Elateeq A.A., Khan Y., Khan J., Khan A., Ali S. (2023). Plant metabolomics: An overview of the role of primary and secondary metabolites against different environmental stress factors. Life.

[18] Reshi Z.A., Ahmad W., Lukatkin A.S., Javed S.B. (2023). From Nature to lab: A review of secondary metabolite biosynthetic pathways, environmental influences, and in vitro approaches. Metabolites.

[19] Tariq H., Asif S., Andleeb A., Hano C., Abbasi B.H. (2023). Flavonoid production: Current trends in plant metabolic engineering and de novo microbial production. Metabolites.

[20] Chen S., Cai R., Liu Z., Cui H., She Z. (2022). Secondary metabolites from mangrove-associated fungi: Source, chemistry and bioactivities. Nat. Prod. Rep..

[21] Hacker K. (2024). The burden of chronic disease. Mayo Clin. Proc. Innov. Qual. Outcomes.

[22] Elshafie H.S., Camele I., Mohamed A.A. (2023). A comprehensive review on the biological, agricultural and pharmaceutical properties of secondary metabolites based-plant origin. Int. J. Mol. Sci..

[23] Marino M., Martini D., Venturi S., Tucci M., Porrini M., Riso P., Del Bo’ C. (2021). An overview of registered clinical trials on glucosinolates and human health: The current situation. Front. Nutr..

[24] Nguyen V.T., Stewart J., Lopez M., Ioannou I., Allais F. (2020). Glucosinolates: Natural occurrence, biosynthesis, accessibility, isolation, structures, and biological activities. Molecules.

[25] Prieto M., López C.J., Simal-Gandara J. (2019). Glucosinolates: Molecular structure, breakdown, genetic, bioavailability, properties and healthy and adverse effects. Adv. Food Nutr. Res..

[26] Ruhee R.T., Suzuki K. (2024). The Immunomodulatory Effects of Sulforaphane in Exercise-Induced Inflammation and Oxidative Stress: A Prospective Nutraceutical. Int. J. Mol. Sci..

[27] Miękus N., Marszałek K., Podlacha M., Iqbal A., Puchalski C., Świergiel A.H. (2020). Health benefits of plant-derived sulfur compounds, glucosinolates, and organosulfur compounds. Molecules.

[28] Ruhee R.T., Roberts L.A., Ma S., Suzuki K. (2020). Organosulfur compounds: A review of their anti-inflammatory effects in human health. Front. Nutr..

[29] Bahoosh S.R., Shokoohinia Y., Eftekhari M. (2022). Glucosinolates and their hydrolysis products as potential nutraceuticals to combat cytokine storm in SARS-CoV-2. DARU J. Pharm. Sci..

[30] Bousquet J., Anto J., Czarlewski W., Haahtela T., Fonseca S., Iaccarino G., Blain H., Vidal A. (2020). Cabbage and fermented vegetables. Allergy.

[31] Kim H.J., Kwon M.S., Hwang H., Choi H.-S., Lee W., Choi S.-P., Jo H., Hong S.W. (2023). A review of the health benefits of kimchi functional compounds and metabolites. Microbiol. Biotechnol. Lett..

[32] Park K.-Y., Ju J. (2018). Kimchi and its health benefits. Korean Functional Foods.

[33] Lavefve L., Marasini D., Carbonero F. (2019). Microbial ecology of fermented vegetables and non-alcoholic drinks and current knowledge on their impact on human health. Adv. Food Nutr. Res..

[34] Kim S.-A., Joung H., Shin S. (2019). Dietary pattern, dietary total antioxidant capacity, and dyslipidemia in Korean adults. Nutr. J..

[35] Das G., Paramithiotis S., Sivamaruthi B.S., Wijaya C.H., Suharta S., Sanlier N., Shin H.-S., Patra J.K. (2020). Traditional fermented foods with anti-aging effect: A concentric review. Food Res. Int..

[36] An S.-Y., Lee M.S., Jeon J.Y., Ha E.S., Kim T.H., Yoon J.Y., Ok C.-O., Lee H.-K., Hwang W.-S., Choe S.J. (2013). Beneficial effects of fresh and fermented kimchi in prediabetic individuals. Ann. Nutr. Metab..

[37] Soundararajan P., Kim J.S. (2018). Anti-carcinogenic glucosinolates in cruciferous vegetables and their antagonistic effects on prevention of cancers. Molecules.

[38] Maina A., Mureithi M., Kiiru J., Revathi G. (2022). Systemic and Mucosal Concentrations of Nine Cytokines Among Individuals with Neisseria gonorrhoeae infection in Nairobi Kenya. AAS Open Res..

[39] Qudus M.S., Tian M., Sirajuddin S., Liu S., Afaq U., Wali M., Liu J., Pan P., Luo Z., Zhang Q. (2023). The roles of critical pro-inflammatory cytokines in the drive of cytokine storm during SARS-CoV-2 infection. J. Med. Virol..

[40] Chams N., Chams S., Badran R., Shams A., Araji A., Raad M., Mukhopadhyay S., Stroberg E., Duval E.J., Barton L.M. (2020). COVID-19: A multidisciplinary review. Front. Public Health.

[41] Panda S.S., Panda D.S., Dixit R. (2022). Revolutionary Solutions for Comprehensive Assessment of COVID-19 Pandemic. Proceedings of International Conference on Computational Intelligence: ICCI 2021, Online, 27–28 December 2021.

[42] Jarczak D., Nierhaus A. (2022). Cytokine storm—Definition, causes, and implications. Int. J. Mol. Sci..

[43] Ye Q., Wang B., Mao J. (2020). The pathogenesis and treatment of theCytokine Storm’in COVID-19. J. Infect..

[44] Chatenoud L., Ferran C., Bach J.-F. (1991). The anti-CD3-induced syndrome: A consequence of massive in vivo cell activation. Superantigens.

[45] Ferrara J.L. (1993). Cytokine dysregulation as a mechanism of graft versus host disease. Curr. Opin. Immunol..

[46] Fara A., Mitrev Z., Rosalia R.A., Assas B.M. (2020). Cytokine storm and COVID-19: A chronicle of pro-inflammatory cytokines. Open Biol..

[47] Cavalli G., Colafrancesco S., Emmi G., Imazio M., Lopalco G., Maggio M.C., Sota J., Dinarello C.A. (2021). Interleukin 1α: A comprehensive review on the role of IL-1α in the pathogenesis and treatment of autoimmune and inflammatory diseases. Autoimmun. Rev..

[48] Di Paolo N.C., Shayakhmetov D.M. (2016). Interleukin 1α and the inflammatory process. Nat. Immunol..

[49] Kim B., Lee Y., Kim E., Kwak A., Ryoo S., Bae S.H., Azam T., Kim S., Dinarello C.A. (2013). The interleukin-1α precursor is biologically active and is likely a key alarmin in the IL-1 family of cytokines. Front. Immunol..

[50] Al-Qahtani A.A., Alhamlan F.S., Al-Qahtani A.A. (2024). Pro-inflammatory and anti-inflammatory interleukins in infectious diseases: A comprehensive review. Trop. Med. Infect. Dis..

[51] Renu K., Subramaniam M.D., Chakraborty R., Myakala H., Iyer M., Bharathi G., Siva K., Vellingiri B., Gopalakrishnan A.V. (2020). The role of Interleukin-4 in COVID-19 associated male infertility–A hypothesis. J. Reprod. Immunol..

[52] Liu X.-G., Li J., Zheng L.-J., Han B., Huang F. (2020). Interleukin-36 receptor antagonist alleviates airway inflammation in asthma via inhibiting the activation of interleukin-36 pathway. Int. Immunopharmacol..

[53] Clinchy B., Gunnerås M., Håkansson A., Håkansson L. (2006). Production of IL-1Ra by human mononuclear blood cells in vitro: Influence of serum factors. Cytokine.

[54] Ihim S.A., Abubakar S.D., Zian Z., Sasaki T., Saffarioun M., Maleknia S., Azizi G. (2022). Interleukin-18 cytokine in immunity, inflammation, and autoimmunity: Biological role in induction, regulation, and treatment. Front. Immunol..

[55] Kaplanski G. (2018). Interleukin-18: Biological properties and role in disease pathogenesis. Immunol. Rev..

[56] Dinarello C.A. (2013). Overview of the interleukin-1 family of ligands and receptors. Semin. Immunol..

[57] Makaremi S., Asgarzadeh A., Kianfar H., Mohammadnia A., Asghariazar V., Safarzadeh E. (2022). The role of IL-1 family of cytokines and receptors in pathogenesis of COVID-19. Inflamm. Res..

[58] Queen D., Ediriweera C., Liu L. (2019). Function and regulation of IL-36 signaling in inflammatory diseases and cancer development. Front. Cell Dev. Biol..

[59] Ahmed A.A., Ad’hiah A.H. (2022). Interleukin-37 gene polymorphism and susceptibility to coronavirus disease 19 among Iraqi patients. Meta Gene.

[60] Rudloff I., Ung H.K., Dowling J.K., Mansell A., D’Andrea L., Ellisdon A.M., Whisstock J.C., Berger P.J., Nold-Petry C.A., Nold M.F. (2020). Parsing the IL-37-mediated suppression of inflammasome function. Cells.

[61] Su Z., Tao X. (2021). Current understanding of IL-37 in human health and disease. Front. Immunol..

[62] Vaz de Paula C.B., de Azevedo M.L.V., Nagashima S., Martins A.P.C., Malaquias M.A.S., Miggiolaro A.F.R.d.S., da Silva Motta Júnior J., Avelino G., do Carmo L.A.P., Carstens L.B. (2020). IL-4/IL-13 remodeling pathway of COVID-19 lung injury. Sci. Rep..

[63] Cesta M.C., Zippoli M., Marsiglia C., Gavioli E.M., Mantelli F., Allegretti M., Balk R.A. (2022). The role of interleukin-8 in lung inflammation and injury: Implications for the management of COVID-19 and hyperinflammatory acute respiratory distress syndrome. Front. Pharmacol..

[64] Huang H., Liu Y., Xiang J. (2002). Synergistic effect of adoptive T-cell therapy and intratumoral interferon γ-inducible protein-10 transgene expression in treatment of established tumors. Cell. Immunol..

[65] Yadav V., Sharma S., Kumar A., Singh S., Ravichandiran V. (2023). Serratiopeptidase Attenuates Lipopolysaccharide-Induced Vascular Inflammation by Inhibiting the Expression of Monocyte Chemoattractant Protein-1. Curr. Issues Mol. Biol..

[66] Jorgovanovic D., Song M., Wang L., Zhang Y. (2020). Roles of IFN-γ in tumor progression and regression: A review. Biomark. Res..

[67] He Z., Tian H., Xing J., Tang X., Sheng X., Chi H., Zhan W. (2023). Full-length transcriptome sequencing of lymphocytes respond to IFN-γ reveals a Th1-skewed immune response in flounder (*Paralichthys olivaceus*). Fish Shellfish Immunol..

[68] Kandikattu H.K., Venkateshaiah S.U., Kumar S., Mishra A. (2020). IL-15 immunotherapy is a viable strategy for COVID-19. Cytokine Growth Factor Rev..

[69] Wilz S.W. (2021). A clinical trial of IL-15 and IL-21 combination therapy for COVID-19 is warranted. Cytokine Growth Factor Rev..

[70] Malahe S.R.K., den Hartog Y., Rietdijk W.J., van Baarle D., de Kuiper R., Reijerkerk D., Ras A.M., Geers D., Diavatopoulos D.A., Messchendorp A.L. (2023). The role of interleukin-21 in COVID-19 vaccine–induced B cell–mediated immune responses in patients with kidney disease and kidney transplant recipients. Am. J. Transplant..

[71] Zhang Y., Su J. (2023). Interleukin-2 family cytokines: An overview of genes, expression, signaling and functional roles in teleost. Dev. Comp. Immunol..

[72] Yazan A. (2021). Interleukin-2 level for normal people and COVID-19 infection: Is it our concern is COVID-19 infection or interleukin-2 level before the infection. Eurasian J. Med. Oncol..

[73] Chen D., Tang T.-X., Deng H., Yang X.-P., Tang Z.-H. (2021). Interleukin-7 biology and its effects on immune cells: Mediator of generation, differentiation, survival, and homeostasis. Front. Immunol..

[74] Wu Z., McGoogan J.M. (2020). Characteristics of and important lessons from the coronavirus disease 2019 (COVID-19) outbreak in China: Summary of a report of 72 314 cases from the Chinese Center for Disease Control and Prevention. JAMA.

[75] Gupta R., Kumar S. (2020). Ten Weeks of COVID-19 Infection in India-Description and Analysis. SSRN 3576320. https://papers.ssrn.com/sol3/papers.cfm?abstract_id=3576320.

[76] Hu B., Guo H., Zhou P., Shi Z.-L. (2021). Characteristics of SARS-CoV-2 and COVID-19. Nat. Rev. Microbiol..

[77] Rothan H.A., Byrareddy S.N. (2021). The potential threat of multisystem inflammatory syndrome in children during the COVID-19 pandemic. Pediatr. Allergy Immunol..

[78] Chen R.-X., Gong H.-Y., Wang X., Sun M.-H., Ji Y.-F., Tan S.-M., Chen J.-M., Shao J.-W., Liao M. (2023). Zoonotic Hantaviridae with global public health significance. Viruses.

[79] Jarczak D., Nierhaus A. (2023). What Is Cytokine Storm?. Management of Dysregulated Immune Response in the Critically Ill.

[80] Vassiliou A.G., Kotanidou A., Dimopoulou I., Orfanos S.E. (2020). Endothelial damage in acute respiratory distress syndrome. Int. J. Mol. Sci..

[81] Van Paassen J., Vos J.S., Hoekstra E.M., Neumann K.M., Boot P.C., Arbous S.M. (2020). Corticosteroid use in COVID-19 patients: A systematic review and meta-analysis on clinical outcomes. Crit. Care.

[82] Belletti A., Campochiaro C., Marmiere M., Likhvantsev V., Yavorovskiy A., Dagna L., Landoni G., Zangrillo A., Hajjar L.A. (2021). Efficacy and safety of IL-6 inhibitors in patients with COVID-19 pneumonia: A systematic review and meta-analysis of multicentre, randomized trials. Ann. Intensive Care.

[83] Zhang X., Shang L., Fan G., Gu X., Xu J., Wang Y., Huang L., Cao B. (2022). The efficacy and safety of janus kinase inhibitors for patients with COVID-19: A living systematic review and meta-analysis. Front. Med..

[84] Bajwah S., Wilcock A., Towers R., Costantini M., Bausewein C., Simon S.T., Bendstrup E., Prentice W., Johnson M.J., Currow D.C. (2020). Managing the supportive care needs of those affected by COVID-19. Eur. Respir. J..

[85] Alipour Z., Zarezadeh S., Ghotbi-Ravandi A.A. (2023). The potential of anti-coronavirus plant secondary metabolites in COVID-19 drug discovery as an alternative to repurposed drugs: A review. Planta Medica.

[86] López-Chillón M.T., Carazo-Díaz C., Prieto-Merino D., Zafrilla P., Moreno D.A., Villaño D. (2019). Effects of long-term consumption of broccoli sprouts on inflammatory markers in overweight subjects. Clin. Nutr..

[87] Chen R., Wang X.-J., Zhang Y.-Y., Xing Y., Yang L., Ni H., Li H.-H. (2019). Simultaneous extraction and separation of oil, proteins, and glucosinolates from Moringa oleifera seeds. Food Chem..

[88] Tetteh O.N.A., Ulrichs C., Huyskens-Keil S., Mewis I., Amaglo N.K., Oduro I.N., Adarkwah C., Obeng-Ofori D., Förster N. (2019). Effects of harvest techniques and drying methods on the stability of glucosinolates in Moringa oleifera leaves during post-harvest. Sci. Hortic..

[89] Brunelli D., Tavecchio M., Falcioni C., Frapolli R., Erba E., Iori R., Rollin P., Barillari J., Manzotti C., Morazzoni P. (2010). The isothiocyanate produced from glucomoringin inhibits NF-kB and reduces myeloma growth in nude mice in vivo. Biochem. Pharmacol..

[90] Hiraga Y., Ara T., Sato N., Akimoto N., Sugiyama K., Suzuki H., Kera K. (2021). Metabolic analysis of unripe papaya (*Carica papaya* L.) to promote its utilization as a functional food. Biosci. Biotechnol. Biochem..

[91] Santana L.F., Inada A.C., Espirito Santo B.L.S.d., Filiú W.F., Pott A., Alves F.M., Guimarães R.d.C.A., Freitas K.d.C., Hiane P.A. (2019). Nutraceutical potential of Carica papaya in metabolic syndrome. Nutrients.

[92] Williams D.J., Pun S., Chaliha M., Scheelings P., O’Hare T. (2013). An unusual combination in papaya (*Carica papaya*): The good (glucosinolates) and the bad (cyanogenic glycosides). J. Food Compos. Anal..

[93] Kim S.-H., Ochar K., Iwar K., Lee Y.-J., Kang H.J., Na Y.-W. (2024). Variations of Major Glucosinolates in Diverse Chinese Cabbage (*Brassica rapa* ssp. *pekinensis*) Germplasm as Analyzed by UPLC-ESI-MS/MS. Int. J. Mol. Sci..

[94] Kim S.-H., Lee G.-A., Subramanian P., Hahn B.-S. (2023). Quantification and Diversity Analyses of Major Glucosinolates in Conserved Chinese Cabbage (*Brassica rapa* L. ssp. *pekinensis*) Germplasms. Foods.

[95] Connolly E.L., Sim M., Travica N., Marx W., Beasy G., Lynch G.S., Bondonno C.P., Lewis J.R., Hodgson J.M., Blekkenhorst L.C. (2021). Glucosinolates from cruciferous vegetables and their potential role in chronic disease: Investigating the preclinical and clinical evidence. Front. Pharmacol..

[96] Burčul F., Ivana G.M., Mila R., Patrick R., Ivica B. (2018). Isothiocyanates: Cholinesterase inhibiting, antioxidant, and anti-inflammatory activity. Enzyme Inhib. Med. Chem..

[97] Kamal R.M., Abdull Razis A.F., Mohd Sukri N.S., Perimal E.K., Ahmad H., Patrick R., Djedaini-Pilard F., Mazzon E., Rigaud S. (2022). Beneficial health effects of glucosinolates-derived isothiocyanates on cardiovascular and neurodegenerative diseases. Molecules.

[98] Ghaffari M. (2021). Herbal Remedies for Management of COVID-19 Induced Myocarditis. Suntext Rev. Cardiovasc. Sci..

[99] Sapra L., Bhardwaj A., Azam Z., Madhry D., Verma B., Rathore S., Srivastava R.K. (2021). Phytotherapy for treatment of cytokine storm in COVID-19. Front. Biosci.-Landmark.

[100] Savant S., Srinivasan S., Kruthiventi A.K. (2021). Potential nutraceuticals for COVID-19. Nutr. Diet. Suppl..

[101] Bousquet J., Le Moing V., Blain H., Czarlewski W., Zuberbier T., De La Torre R., Lozano N.P., Reynes J., Bedbrook A., Cristol J.-P. (2021). Efficacy of broccoli and glucoraphanin in COVID-19: From hypothesis to proof-of-concept with three experimental clinical cases. World Allergy Organ. J..

[102] Giacoppo S., Galuppo M., Iori R., De Nicola G.R., Cassata G., Bramanti P., Mazzon E. (2013). Protective role of (RS)-glucoraphanin bioactivated with myrosinase in an experimental model of multiple sclerosis. CNS Neurosci. Ther..

[103] Esteve M. (2020). Mechanisms underlying biological effects of cruciferous glucosinolate-derived isothiocyanates/indoles: A focus on metabolic syndrome. Front. Nutr..

[104] Caglayan B., Kilic E., Dalay A., Altunay S., Tuzcu M., Erten F., Orhan C., Gunal M.Y., Yulug B., Juturu V. (2019). Allyl isothiocyanate attenuates oxidative stress and inflammation by modulating *Nrf2*/HO-1 and NF-κB pathways in traumatic brain injury in mice. Mol. Biol. Rep..

[105] Subedi L., Venkatesan R., Kim S.Y. (2017). Neuroprotective and anti-inflammatory activities of allyl isothiocyanate through attenuation of JNK/NF-κB/TNF-α signaling. Int. J. Mol. Sci..

[106] Cheung K.L., Kong A.-N. (2010). Molecular targets of dietary phenethyl isothiocyanate and sulforaphane for cancer chemoprevention. AAPS J..

[107] Ernst I.M., Palani K., Esatbeyoglu T., Schwarz K., Rimbach G. (2013). Synthesis and *Nrf2*-inducing activity of the isothiocyanates iberverin, iberin and cheirolin. Pharmacol. Res..

[108] Gasparello J., D’Aversa E., Papi C., Gambari L., Grigolo B., Borgatti M., Finotti A., Gambari R. (2021). Sulforaphane inhibits the expression of interleukin-6 and interleukin-8 induced in bronchial epithelial IB3-1 cells by exposure to the SARS-CoV-2 Spike protein. Phytomedicine.

[109] Jaafaru M.S., Razis A.F.A. (2022). Sulfur compounds. Bioactive Food Components Activity in Mechanistic Approach.

[110] Tran H.T., Herz C., Ruf P., Stetter R., Lamy E. (2018). Human T2R38 bitter taste receptor expression in resting and activated lymphocytes. Front. Immunol..

[111] Jaafaru M.S., Abd Karim N.A., Enas M.E., Rollin P., Mazzon E., Abdull Razis A.F. (2018). Protective effect of glucosinolates hydrolytic products in neurodegenerative diseases (NDDs). Nutrients.

[112] Wu Q., Zhou H.-J., Sheng J., Su L.-Y., Tian Y. (2023). Extraction, structural properties, and bioactivities of Moringa (*Moringa oleifera* Lam.) isothiocyanates: A review. Food Biosci..

[113] Su C.-M., Wang L., Yoo D. (2021). Activation of NF-κB and induction of proinflammatory cytokine expressions mediated by ORF7a protein of SARS-CoV-2. Sci. Rep..

[114] Negi G., Kumar A., S Sharma S. (2011). *Nrf2* and NF-κB modulation by sulforaphane counteracts multiple manifestations of diabetic neuropathy in rats and high glucose-induced changes. Curr. Neurovasc. Res..

[115] Xu C., Shen G., Chen C., Gélinas C., Kong A.-N.T. (2005). Suppression of NF-κB and NF-κB-regulated gene expression by sulforaphane and PEITC through IκBα, IKK pathway in human prostate cancer PC-3 cells. Oncogene.

[116] Pant T., Uche N., Juric M., Zielonka J., Bai X. (2024). Regulation of immunomodulatory networks by *Nrf2*-activation in immune cells: Redox control and therapeutic potential in inflammatory diseases. Redox Biol..

[117] Deramaudt T.B., Ali M., Vinit S., Bonay M. (2020). Sulforaphane reduces intracellular survival of Staphylococcus aureus in macrophages through inhibition of JNK and p38 MAPK-induced inflammation. Int. J. Mol. Med..

[118] Tarozzi A., Angeloni C., Malaguti M., Morroni F., Hrelia S., Hrelia P. (2013). Sulforaphane as a potential protective phytochemical against neurodegenerative diseases. Oxidative Med. Cell. Longev..

[119] Olagnier D., Farahani E., Thyrsted J., Blay-Cadanet J., Herengt A., Idorn M., Hait A., Hernaez B., Knudsen A., Iversen M.B. (2020). SARS-CoV2-mediated suppression of *NRF2*-signaling reveals potent antiviral and anti-inflammatory activity of 4-octyl-itaconate and dimethyl fumarate. Nat. Commun..

[120] Kiser C., Gonul C.P., Olcum M., Genc S. (2021). Inhibitory effects of sulforaphane on NLRP3 inflammasome activation. Mol. Immunol..

[121] Gasparello J., Marzaro G., Papi C., Gentili V., Rizzo R., Zurlo M., Scapoli C., Finotti A., Gambari R. (2023). Effects of Sulforaphane on SARS-CoV-2 infection and NF-κB dependent expression of genes involved in the COVID-19 ‘cytokine storm’. Int. J. Mol. Med..

[122] Ordonez A.A., Bullen C.K., Villabona-Rueda A.F., Thompson E.A., Turner M.L., Merino V.F., Yan Y., Kim J., Davis S.L., Komm O. (2022). Sulforaphane exhibits antiviral activity against pandemic SARS-CoV-2 and seasonal HCoV-OC43 coronaviruses in vitro and in mice. Commun. Biol..

[123] Kow C.S., Ramachandram D.S., Hasan S.S. (2022). Use of sulforaphane in COVID-19: Clinical trials are needed. Mol. Immunol..

[124] Pan P., Shen M., Yu Z., Ge W., Chen K., Tian M., Xiao F., Wang Z., Wang J., Jia Y. (2021). SARS-CoV-2 N protein promotes NLRP3 inflammasome activation to induce hyperinflammation. Nat. Commun..

[125] Tufekci K.U., Ercan I., Isci K.B., Olcum M., Tastan B., Gonul C.P., Genc K., Genc S. (2021). Sulforaphane inhibits NLRP3 inflammasome activation in microglia through *Nrf2*-mediated miRNA alteration. Immunol. Lett..

[126] Chen Z., Du R., Cooper L., Achi J.G., Dong M., Ran Y., Zhang J., Zhan P., Rong L., Cui Q. (2023). Sulforaphane is a reversible covalent inhibitor of 3-chymotrypsin-like protease of SARS-CoV-2. J. Med. Virol..

[127] Hoch C.C., Shoykhet M., Weiser T., Griesbaum L., Petry J., Hachani K., Multhoff G., Dezfouli A.B., Wollenberg B. (2024). Isothiocyanates in medicine: A comprehensive review on phenylethyl-, allyl-, and benzyl-isothiocyanates. Pharmacol. Res..

[128] Castro V., Carpena M., Fraga-Corral M., Lopez-Soria A., Garcia-Perez P., Barral-Martinez M., Perez-Gregorio R., Cao H., Simal-Gandara J., Prieto M. (2023). Sulfur-containing compounds from plants. Natural Secondary Metabolites: From Nature, through Science, to Industry.

[129] Sita G., Hrelia P., Tarozzi A., Morroni F. (2016). Isothiocyanates are promising compounds against oxidative stress, neuroinflammation and cell death that may benefit neurodegeneration in Parkinson’s disease. Int. J. Mol. Sci..

[130] Fuentes F., Paredes-Gonzalez X., Kong A.-N.T. (2015). Dietary glucosinolates sulforaphane, phenethyl isothiocyanate, indole-3-carbinol/3, 3′-diindolylmethane: Antioxidative stress/inflammation, *Nrf2*, epigenetics/epigenomics and in vivo cancer chemopreventive efficacy. Curr. Pharmacol. Rep..

[131] Ramirez C.N., Li W., Zhang C., Wu R., Su S., Wang C., Gao L., Yin R., Kong A.-N. (2018). In vitro-in vivo dose response of ursolic acid, sulforaphane, PEITC, and curcumin in cancer prevention. AAPS J..

[132] Baskar V., Krishnan M., Rajakumar G., Hariram Nile S., Thiruvengadam M. (2023). Modulation of Expression Levels of Various Cytokines and Inflammatory Responses by Glucosinolate Derivatives. Curr. Top. Med. Chem..

[133] Aslam M.S., Ahmad M.A. (2023). Multidisciplinary Applications of Natural Science for Drug Discovery and Integrative Medicine.

[134] Okamoto T., Akita N., Nagai M., Hayashi T., Suzuki K. (2014). 6-Methylsulfinylhexyl isothiocyanate modulates endothelial cell function and suppresses leukocyte adhesion. J. Nat. Med..

[135] Chen J., Uto T., Tanigawa S., Yamada-Kato T., Fujii M., Hou D.-X. (2010). Microarray-based determination of anti-inflammatory genes targeted by 6-(methylsulfinyl) hexyl isothiocyanate in macrophages. Exp. Ther. Med..

[136] Kobayashi E.H., Suzuki T., Funayama R., Nagashima T., Hayashi M., Sekine H., Tanaka N., Moriguchi T., Motohashi H., Nakayama K. (2016). *Nrf2* suppresses macrophage inflammatory response by blocking proinflammatory cytokine transcription. Nat. Commun..

[137] Yang L., Xie X., Tu Z., Fu J., Xu D., Zhou Y. (2021). The signal pathways and treatment of cytokine storm in COVID-19. Signal Transduct. Target. Ther..

[138] Pagnotta E., Ugolini L., Matteo R., Righetti L. (2022). Bioactive compounds from Eruca sativa seeds. Encyclopedia.

[139] Melchini A., Traka M.H. (2010). Biological profile of erucin: A new promising anticancer agent from cruciferous vegetables. Toxins.

[140] Wagner A.E., Sturm C., Piegholdt S., Wolf I.M., Esatbeyoglu T., De Nicola G.R., Iori R., Rimbach G. (2015). Myrosinase-treated glucoerucin is a potent inducer of the *Nrf2* target gene heme oxygenase 1—Studies in cultured HT-29 cells and mice. J. Nutr. Biochem..

[141] Maresca D.C., Conte L., Romano B., Ianaro A., Ercolano G. (2022). Antiproliferative and Proapoptotic Effects of Erucin, a Diet-Derived H2S Donor, on Human Melanoma Cells. Antioxidants.

[142] Hanlon N. (2008). The Chemopreventive Potential of Sulforaphane and Erucin.

[143] Genah S., Ciccone V., Filippelli A., Simonis V., Martelli A., Piragine E., Pagnotta E., Pecchioni N., Calderone V., Morbidelli L. (2024). Erucin, a natural isothiocyanate, exerts pro-angiogenic properties in cultured endothelial cells and reverts angiogenic impairment induced by high glucose. Phytother. Res..

[144] Singh S., Singh G., Attri S., Kaur P., Rashid F., Bedi N., Haque S., Janahi E.M., Arora S. (2023). Development and optimization of nanoparticles loaded with erucin, a dietary isothiocyanate isolated from Eruca sativa: Antioxidant and antiproliferative activities in ehrlich-ascites carcinoma cell line. Front. Pharmacol..

[145] Cho H.J., Lee K.W., Park J.H.Y. (2013). Erucin exerts anti-inflammatory properties in murine macrophages and mouse skin: Possible mediation through the inhibition of NFκB signaling. Int. J. Mol. Sci..

[146] Yehuda H., Soroka Y., Zlotkin-Frušić M., Gilhar A., Milner Y., Tamir S. (2012). Isothiocyanates inhibit psoriasis-related proinflammatory factors in human skin. Inflamm. Res..

[147] Hosokawa Y., Hosokawa I., Shimoyama M., Fujii A., Sato J., Kadena K., Ozaki K., Hosaka K. (2022). The anti-inflammatory effects of iberin on TNF-α-stimulated human oral epithelial cells: In vitro research. Biomedicines.

[148] Mazumder A., Dwivedi A., Du Plessis J. (2016). Sinigrin and its therapeutic benefits. Molecules.

[149] Subedi L., Lee J.H., Yumnam S., Ji E., Kim S.Y. (2019). Anti-inflammatory effect of sulforaphane on LPS-activated microglia potentially through JNK/AP-1/NF-κB inhibition and *Nrf2*/HO-1 activation. Cells.

[150] Liu Y., Chin F.W.L., Huang D., Liu S.-Q., Lu Y. (2024). The thermal degradation of glucomoringin and changes of phenolic compounds in moringa seed kernels during different degrees of roasting. Food Chem..

[151] Manjunath S.H., Nataraj P., Swamy V.H., Sugur K., Dey S.K., Ranganathan V., Daniel S., Leihang Z., Sharon V., Chandrashekharappa S. (2023). Development of Moringa oleifera as functional food targeting *NRF2* signaling: Antioxidant and anti-inflammatory activity in experimental model systems. Food Funct..

[152] Chiricosta L., Gugliandolo A., Diomede F., Pizzicannella J., Trubiani O., Iori R., Tardiolo G., Guarnieri S., Bramanti P., Mazzon E. (2019). Moringin pretreatment inhibits the expression of genes involved in mitophagy in the stem cell of the human periodontal ligament. Molecules.

[153] Zhang T., Zhao L., Xu M., Jiang P., Zhang K. (2024). Moringin alleviates DSS-induced ulcerative colitis in mice by regulating *Nrf2*/NF-κB pathway and PI3K/AKT/mTOR pathway. Int. Immunopharmacol..

[154] Giacoppo S., Rajan T.S., De Nicola G.R., Iori R., Rollin P., Bramanti P., Mazzon E. (2017). The isothiocyanate isolated from Moringa oleifera shows potent anti-inflammatory activity in the treatment of murine subacute Parkinson’s disease. Rejuvenation Res..

